# Significant Reduction of Antibiotic Use in the Community after a Nationwide Campaign in France, 2002–2007

**DOI:** 10.1371/journal.pmed.1000084

**Published:** 2009-06-02

**Authors:** Elifsu Sabuncu, Julie David, Claire Bernède-Bauduin, Sophie Pépin, Michel Leroy, Pierre-Yves Boëlle, Laurence Watier, Didier Guillemot

**Affiliations:** 1 INSERM, U657, Paris, France; 2 Institut Pasteur, Pharmacoépidémiologie et Maladies Infectieuses, Paris, France; 3 Caisse Nationale d'Assurance Maladie des Travailleurs Salariés, Paris, France; 4 Régime Social des Indépendants, La Plaine-Saint-Denis, France; 5 INSERM, U707, Paris, France; 6 Faculté de Médecine Saint Antoine, Université Pierre et Marie Curie, Paris, France; 7 INSERM, U780, Villejuif, France; 8 Université Paris-Sud 11, IFR69, Le Kremlin-Bicêtre, France; 9 Faculté de Médecine Paris Ile-de-France Ouest, Université Versailles Saint-Quentin, Versailles, France; 10 Département de médecine aigüe, Hôpital Universitaire Raymond-Poincaré, Assistance Publique-Hôpitaux de Paris, Garches, France; Emory University, United States of America

## Abstract

**Background:**

Overuse of antibiotics is the main force driving the emergence and dissemination of bacterial resistance in the community. France consumes more antibiotics and has the highest rate of beta-lactam resistance in *Streptococcus pneumoniae* than any other European country. In 2001, the government initiated “Keep Antibiotics Working”; the program's main component was a campaign entitled “Les antibiotiques c'est pas automatique” (“Antibiotics are not automatic”) launched in 2002. We report the evaluation of this campaign by analyzing the evolution of outpatient antibiotic use in France 2000–2007, according to therapeutic class and geographic and age-group patterns.

**Methods and Findings:**

This evaluation is based on 2000–2007 data, including 453,407,458 individual reimbursement data records and incidence of flu-like syndromes (FLSs). Data were obtained from the computerized French National Health Insurance database and provided by the French Sentinel Network. As compared to the preintervention period (2000–2002), the total number of antibiotic prescriptions per 100 inhabitants, adjusted for FLS frequency during the winter season, changed by −26.5% (95% confidence interval [CI] −33.5% to −19.6%) over 5 years. The decline occurred in all 22 regions of France and affected all antibiotic therapeutic classes except quinolones. The greatest decrease, −35.8% (95% CI −48.3% to −23.2%), was observed among young children aged 6–15 years. A significant change of −45% in the relationship between the incidence of flu-like syndromes and antibiotic prescriptions was observed.

**Conclusions:**

The French national campaign was associated with a marked reduction of unnecessary antibiotic prescriptions, particularly in children. This study provides a useful method for assessing public-health strategies designed to reduce antibiotic use.

## Abstract 2

Didier Guillemot and colleagues describe the evaluation of a nationwide programme in France aimed at decreasing unnecessary outpatient prescriptions for antibiotics. The campaign was successful, particularly in reducing prescriptions for children.

## Editors' Summary

**Background:**

In 1928, Alexander Fleming discovered penicillin, the first antibiotic (a drug that kills bacteria). By the early 1940s, large amounts of penicillin could be made and, in the following decades, several other classes of powerful antibiotics were discovered. For a time, it looked like bacteria and the diseases that they cause had been defeated. But bacteria rapidly became resistant to these wonder drugs and nowadays, antibiotic resistance is a pressing public-health concern. Almost every type of disease-causing bacteria has developed resistance to one or more antibiotic in clinical use, and multidrug-resistant bacteria are causing outbreaks of potentially fatal diseases in hospitals and in the community. For example, multidrug-resistant *Streptococcus pneumoniae* (multidrug-resistant pneumococci or MRP) is now very common. *S. pneumoniae* colonize the nose and throat (the upper respiratory tract) and can cause diseases that range from mild ear infections to life-threatening pneumonia, particularly in young children and elderly people.

**Why Was This Study Done?:**

For years, doctors have been prescribing (and patients have been demanding) antibiotics for viral respiratory infections (VRIs) such as colds and flu even though antibiotics do not cure viral infections. This overuse of antibiotics has been the main driving force in the spread of MRP. Thus, the highest rate of *S. pneumoniae* antibiotic resistance in Europe occurs in France, which has one of the highest rates of antibiotic consumption in the world. In 2001 France initiated “le plan national pour préserver l'efficacité des antibiotiques” to reduce the inappropriate use of antibiotics, particularly for the treatment of VRIs among children. The main component of the program was the “Antibiotiques c'est pas automatique” (“Antibiotics are not automatic”) campaign, which ran from 2002 to 2007 during the winter months when VRIs mainly occur. The campaign included an educational campaign for health care workers, the promotion of rapid tests for diagnosis of streptococcal infections, and a public information campaign about VRIs and about antibiotic resistance. In this study, the researchers evaluate the campaign by analyzing outpatient antibiotic use throughout France from 2000 to 2007.

**What Did the Researchers Do and Find?:**

The researchers obtained information about antibiotic prescriptions and about the occurrence of flu-like illnesses during the study period from the French National Health Insurance database and national disease surveillance system, respectively. After adjusting for variations in the frequency of flu-like illnesses, compared to the preintervention period (2000–2002), the number of antibiotic prescriptions per 100 inhabitants decreased by a quarter over the five winters of the “Antibiotics are not automatic” campaign. The use of all major antibiotic classes except quinolones decreased in all 22 regions of France. Thus, whereas in 2000, more than 70 prescriptions per 100 inhabitants were issued during the winter in 15 regions, by 2006/7, no regions exceeded this prescription rate. The greatest decrease in prescription rate (a decrease of more than a third by 2006/7) was among children aged 6–15 years. Finally, although the rates of antibiotic prescriptions reflected the rates of flu-like illness throughout the campaign, by 2006/7 this relationship was much weaker, which suggests that fewer antibiotics were being prescribed for VRIs.

**What Do These Findings Mean?:**

These findings indicate that the “Antibiotics are not automatic” campaign was associated with a reduction in antibiotic prescriptions, particularly in children. Because the whole French population was exposed to the campaign, these findings do not prove that the campaign actually caused the reduction in antibiotic prescriptions. The observed decrease might have been caused by other initiatives in France or elsewhere or by the introduction of a *S. pneumoniae* vaccine during the study period, for example. However, an independent survey indicated that fewer members of the public expected an antibiotic prescription for a VRI at the end of the campaign than at the start, that more people knew that antibiotics only kill bacteria, and that doctors were more confident about not prescribing antibiotics for VRIs. Thus, campaigns like “Antibiotics are not automatic” may be a promising way to reduce the overuse of antibiotics and to slow the spread of antibiotic resistance until new classes of effective antibiotics are developed.

**Additional Information:**

Please access these Web sites via the online version of this summary at http://dx.doi.org/10.1371/journal.pmed.1000084.

- This study is further discussed in a *PLoS Medicine* Perspective by Stephen Harbarth and Benedikt Huttner
- The Bugs and Drugs Web site from the UK National electronic Library of Infection provides information about antibiotic resistance and links to other resources
- The US National Institute of Allergy and Infectious Diseases provides information on antimicrobial drug resistance and on pneumococcal pneumonia
- The US Centers for Disease Control and Prevention also have information on antibiotic resistance (in English and Spanish)
- The European Surveillance of Antimicrobial Consumption Web site provides information on antibiotic consumption in European countries
- Les antibiotiques c'est pas automatique provides information about the “Antibiotics are not automatic” campaign (in French)
- Information on the Plan National pour Pérserver l'efficacité des antibiotiques is also available (in French)

## Introduction

The emergence and the dissemination of drug-resistant bacterial strains make treatment decisions challenging and may be associated with treatment failures. This phenomenon has become a major public health issue.

*Streptococcus pneumoniae* is the most commonly identified bacterial cause of community-acquired invasive infections and pneumonia [1],[2]. Multidrug-resistant pneumococci (MRP) are now ubiquitous, despite the recent availability and now wide use of 7-valent pneumococcal conjugate vaccine (7-PCV) in developed countries, with more infections being caused by nonvaccine serotypes [3].

Very few new antimicrobial drugs are expected to become available in the near future, and several studies support the notion that antibiotic consumption is a key driving force in the rate (number of resistant isolates/total number of isolates) of beta-lactam–resistant pneumococci [4] and in the dissemination of MRP [5]–[7]. Furthermore, there is some evidence that decreasing antibiotic use can lower MRP rates [8]. For instance, the highest MRP rates are reported in southern Europe [9], southeastern Asia [10], and North America [11], where antibiotic consumption is generally higher than in northern Europe [12]. Thus, many countries have undertaken public health programs to optimize antibiotic prescriptions in the community [13].

In the early 2000s, France, a country with nearly 62,000,000 inhabitants and nearly 54,000 general practitioners, faced growing problems with MRP, with >50% of strains showing decreased penicillin G susceptibility. France was also identified as the country with the highest antibiotic consumption in Europe [12] and one of the highest antimicrobial users worldwide. Thus, the French government initiated a long-term nationwide campaign to reduce antibiotic overuse and control the dissemination of resistant bacteria in the community. The national program, named “Keep Antibiotics Working,” was launched in 2001, targeting both the general public and health care professionals, to encourage surveillance of antibiotic use and resistance and to promote better-targeted antibiotic use. Since 2002, a public service campaign entitled “Les antibiotiques c'est pas automatique” (“Antibiotics are not automatic”) is launched each winter with the primary goal of decreasing prescriptions, particularly during the viral respiratory infection (VRI) epidemic season and among children, for whom >40% of the prescriptions are written [14]–[16].

In this article we evaluate the effectiveness of these campaigns by analyzing the evolution of outpatient antibiotic use in France from 2000 to 2007, for each therapeutic class, as well as by geographic and age-group patterns. We performed a time-series analysis and accounted for flu-like syndrome (FLS) variations.

## Methods

### The Campaign (“Antibiotics Are Not Automatic”)

In 2002 the French National Health Insurance (NHI) launched a long-term nationwide campaign in the community. From its conception, the aim of the campaign was to decrease total antibiotic use in the community by 25%, targeting predominantly VRIs in young children. An extensive information dissemination campaign was developed with the central theme “Antibiotics are not automatic.” Each winter (October–March) it is relaunched, as higher levels of infections and prescriptions occur during this period. A description of the campaign is detailed in Text S1.

### Definitions and Data Sources

The NHI program covers all medical care provided by physicians in private practice, community clinics, and hospitals. Patients pay health service fees, which are refunded by the NHI. In France, everyone, even those with low or no income, are covered by the NHI program. We used anonymous computerized individual data from two main NHI agencies (General Scheme and Social Scheme), which cover salaried workers and the self-employed (≥91% of the French population), to access all antibiotics prescribed, dispensed by outpatient pharmacies, and reimbursed by NHI from 2000 to 2007 (beginning week 27 [July] 2000, ending week 13 [March] 2007). Each drug approved by the French Drug Agency is assigned a unique seven-digit code that enables identification of a particular product and its specific dosage, package size, formulation, and manufacturer. Each file contains this drug-related information, prescription date, patient's sex, year of birth, and region of residence.

Weekly FLS incidence was provided by the French Sentinel Network [17] (see http://sentiweb.org/). According to the French Sentinel Network, FLS is defined as the combination of the following clinical symptoms: sudden onset fever ≥39°C, myalgia, and respiratory symptoms such as dyspnea and/or cough. Demographic data were obtained from the French National Institute for Statistics and Economic Studies (INSEE, http://www.insee.fr).

We included the European part of France (henceforth referred to as France), which accounts for 83% of French territory and 96% of its population. For each of France's 22 administrative regions, reimbursement data were extrapolated to 100% of the regional population by dividing the number of prescriptions by the corresponding region's NHI coverage rate. The results are presented as the weekly rate of antibiotic prescriptions per 100 inhabitants.

The study concerns antibiotics for systemic use (anatomical therapeutic class [ATC] code J01) in the community. Antibiotics were divided into six categories according to the ATC classification: penicillins, cephalosporins, macrolides, quinolones, cyclines, and “other.”

### Statistical Analysis

Our analysis describes the crude number of antibiotic prescriptions and FLS incidence over time, overall, and by region, antibiotic class, and age group (0–5, 6–15, >15 y). We focused on the period targeted by the public service campaign (October–March); more specifically, we examined the truncated series corresponding to a 26-wk period starting at week 40 of year *n* and finishing at week 13 of year *n*+1. We conducted time-series analyses to quantitatively evaluate the impact of the successive, annual campaigns on antibiotic consumption. We used intervention models [18]–[20] that allowed us to estimate crude and FLS-adjusted effects of the campaign.

The intervention models were built in two steps. For the first step, we chose the 2000–2002 period preceding the first campaign, i.e., starting at week 27 in 2000 and ending at week 40 in 2002, as the baseline and fitted a model to the series of antibiotic prescriptions accounting for seasonal variations and underlying autoregressive and moving average processes. As in other southern and eastern European countries [21], antibiotic consumption in France shows marked yearly seasonal fluctuations; therefore we first estimated a periodic trigonometric function and removed it from the truncated series so that the residual series was in stationary mode. We then fitted an autoregressive and moving average (ARMA) model [22] on the residual series, leaving white noise residual series.

For the second step, we assumed that observations before and after the campaign were drawn from the same ARMA model with changes only in the mean. Following this assumption, we removed the previously estimated periodic trigonometric function from the whole antibiotic prescription series (2000–2007) and added five dummy variables, one for each annual campaign, to the ARMA model identified in step 1 so that only the mean could change. We quantified the impact of each campaign as the average relative change for the period (from winter 2002–2003 to winter 2006–2007) compared with baseline (2000–2002); that is, the ratio of the observed change over the expected change predicted from the model had there been no campaign. The same procedure was repeated after dividing the population into three age categories (<6, 6–15, and >15 y) as well as considering data of the summer period (April–September) and data of the entire year (October–September).

Finally, we further adjusted the intervention model for the whole population for FLS incidence. We tested the hypothesis of a different link between antibiotic consumption and FLS incidence before and after the start of the 2002 campaign by introducing an interaction term in the model.

Analyses were performed using SAS version 9.1 (SAS Institute, http://www.sas.com/). All statistical tests were two-tailed; a *p*-value of less than 0.05 was considered significant.

## Results

### Description of the Data

Between July 2000 and March 2007, a total of 453,407,458 individual antibiotic prescriptions were reimbursed by the NHI; all files were processed for analysis. Because prescription numbers were similar for men and women (unpublished data), we combined their data.

**Figure 1:**
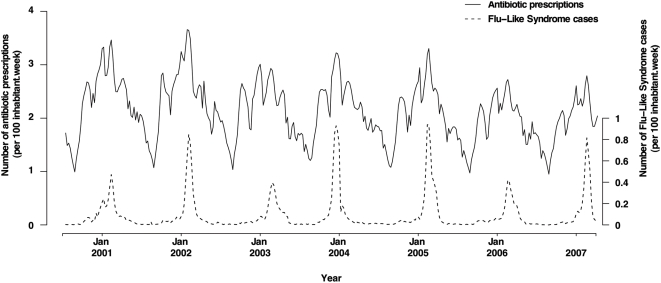
Antibiotic use and flu-like syndromes in France, from July 2000 to March 2007. Weekly totals of antibiotic prescriptions and FLS cases per 100 inhabitants plotted against time.

Antibiotic prescriptions and FLS fluctuated seasonally, with higher incidence rates during winter months (Figure 1). Maximal values differed from one year to another. The number of antibiotic prescriptions varied between 585,524 (week 33 of 2006) and 2,196,942 (week 3 of 2002) (amplitude ratio 3.8). FLS peaks varied between 396 (winter 2002–2003) and 939 (winter 2004–2005) cases per 100,000 inhabitants (amplitude ratio 2.3). The FLS epidemic began as early as week 45 (third week of November) in the 2003–2004 season and as late as week 4 (fourth week of January) in the 2001–2002 season. All epidemics occurred between week 40 (beginning of October) and week 13 (end of March).

The mean number of prescriptions per 100 inhabitants for the general population was 72.4 for the 2000–2001 and 2001–2002 winters; this figure gradually decreased to 56.6 during the 2006–2007 season (Table 1).

**Table 1:** Mean number of prescriptions between October and March, per 100 inhabitants (percent change compared to 2000–2002).

Antibiotic Class	2000–2002	2002–2003	2003–2004	2004–2005	2005–2006	2006–2007
Penicillins	27.0	21.7 (−19.6)	21.5 (−20.4)	20.7 (−23.6)	20.8 (−23.2)	20.2 (−25.3)
Cephalosporins	16.3	13.6 (−17.0)	14.7 (−10.2)	14.6 (−10.7)	12.5 (−23.7)	12.3 (−24.6)
Macrolides	16.4	14.2 (−13.7)	14.2 (−13.8)	13.9 (−15.3)	12.4 (−24.4)	11.5 (−30.1)
Quinolones	4.2	4.3 (3.2)	4.3 (2.8)	4.8 (14.2)	4.6 (8.6)	4.7 (12.8)
Cyclines	3.1	3.1 (1.0)	3.2 (3.7)	3.2 (3.2)	3.1 (1.1)	3.0 (−3.7)
Other	5.3	8.1 (55.0)	8.5 (62.0)	7.4 (40.6)	5.1 (−3.8)	4.8 (−8.0)
All	72.4	65.1 (−10.1)	66.4 (−8.3)	64.5 (−10.8)	58.4 (−19.3)	56.6 (−21.9)

**Figure 2:**
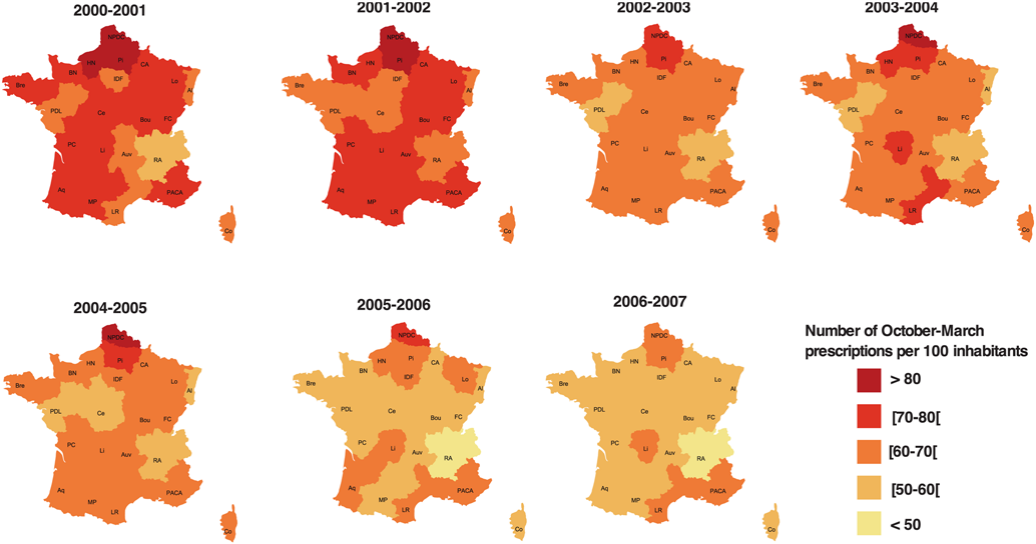
Winter antibiotic prescriptions in France by region, from October 2000 to March 2007. The number of October–March prescriptions is divided by the number of regional inhabitants for the respective year in each of 22 France's regions: Al (Alsace), Aq (Aquitaine), Auv (Auvergne), BN (Basse Normandie), Bou (Bourgogne), Br (Bretagne), CA (Champagne-Ardenne), Ce (Centre), Co (Corse), HN (Haute Normandie), Li (Limousin), Lo (Lorraine), LR (Languedoc-Roussillon), IDF (Ile de France), FC (Franche-Conté), MP (Midi-Pyrénées), NPDC (Nord-Pas de Calais), PACA (Provence-Alpes-Cote d'Azur), PDL (Pays de Loire), PC (Poitou-Charente), Pi (Picardie), RA (Rhones Alpes).

Variations in antibiotic use were observed among France's 22 administrative regions. Antibiotic consumption declined in all regions between 2001–2002 and 2006–2007 (Figure 2). In 2000–2001, 15/22 regions had >70 prescriptions per 100 inhabitants, but none exceeded this level in 2006–2007. The most important reduction was observed in the Centre Region (−28.4%). Among the six administrative regions with the highest average number of prescriptions per 100 inhabitants in 2001–2002 (Nord 91.8, Picardie 83.3, Haute Normandie 80.3, Lorraine 78.7, Champagne-Ardenne 78.7, and Poitou-Charentes 77.3), the decrease in antibiotic use was among the seven highest, with values of −25.2%, −26.1%, −25.6%, −25.2%, −27.1%, and −27.30%, respectively. Antibiotic prescription differences among regions persisted after the campaign but were less dramatic.

Penicillins, cephalosporins, and macrolides were the three most used antibiotic classes at baseline, with 27.0, 16.3, and 16.4 prescriptions per 100 inhabitants; their use also declined the most (changes of −25.3%, −24.6%, and −30.1%, respectively) among all antibiotic classes. Quinolones, which remain a less frequently prescribed antibiotic class (4.2 prescriptions per 100 inhabitants at baseline), was the only class whose use increased (+12.8%) (Table 1).

**Figure 3:**
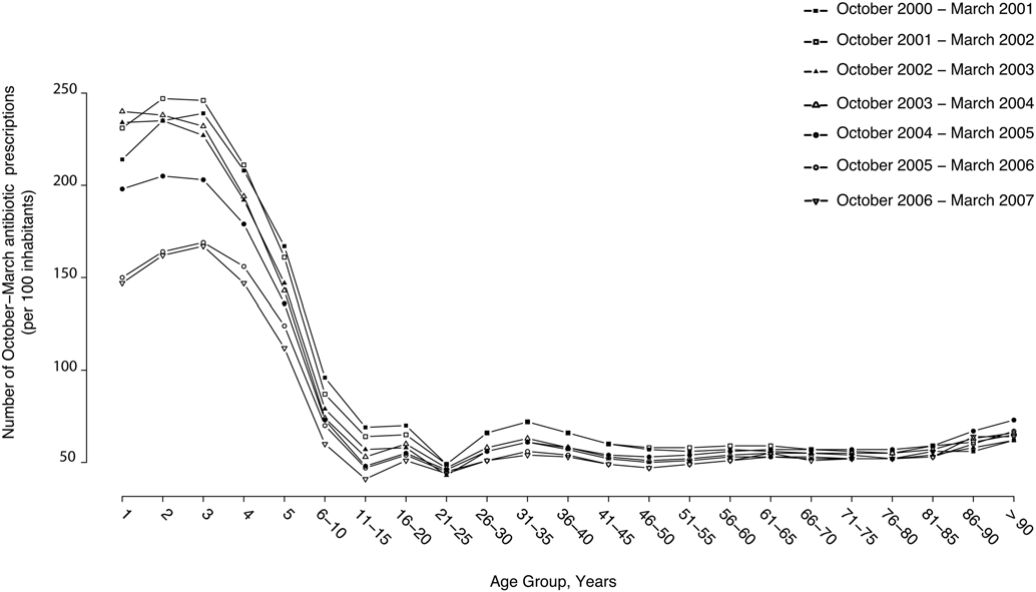
Antibiotic prescriptions in France per 100 inhabitants by age group, from July 2000 to March 2007. The number of October–March prescriptions for each age group for each year is divided by the number of inhabitants of each age group.

For children <6 y old, the average number of prescriptions per 100 children declined from 193.3 in 2001–2002 to 128.7 in 2006–2007. For 2- and 3-y-old children, this figure decreased from ∼250 to ∼150. A substantial reduction of antibiotic use was also observed for children 6–15 y old and young adults 26–35 y old (Figure 3).

### Time-Series Analysis

**Figure 4:**
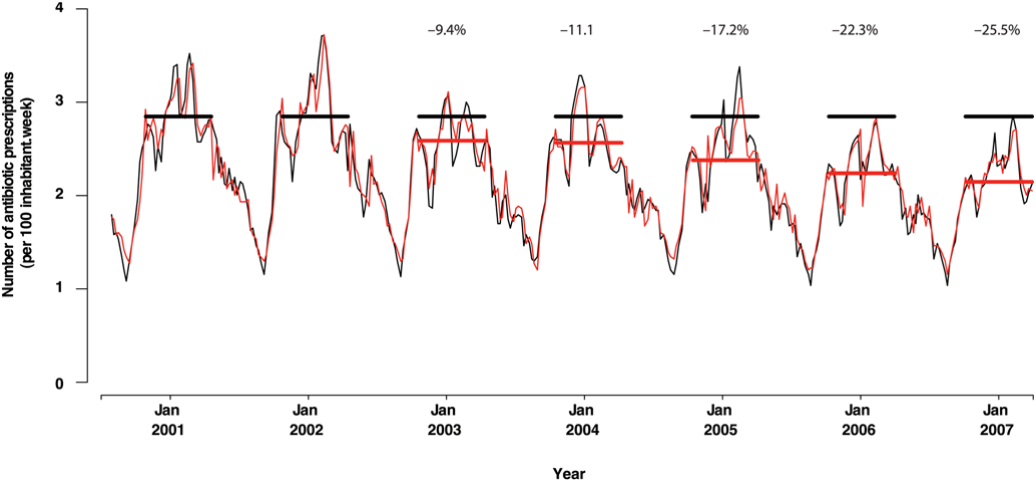
One step ahead forecasts of the interrupted ARMA model and observed antibiotic prescriptions data. October–March horizontal lines indicate the estimated average level by the interrupted ARMA model (in red) and the observed average level without any campaign effect (in black) each winter. The percentages listed above the peaks denote the ratio of change.

Three factors led us to consider 2 y of preintervention data as sufficient to correctly estimate modifications after 2002: (1) the stability of the estimated ARMA model parameters for the 2000–2007 period as compared with 2000–2002, (2) the assessment of Gaussian white noise residuals, and (3) the good fit of the one-step-ahead forecasts of the interrupted ARMA model with observed antibiotic prescriptions (Figure 4). Estimations of average relative change, with corresponding 95% confidence intervals (CIs), are reported in Table 2. The first campaign yielded immediate returns, with statistically significant estimated reductions for the whole population and for inhabitants >15 y old (−9.8% [95% CI −14.9% to −4.7%] and –12.5% [95% CI −16.8% to −8.1%], respectively). The evolution of this change differed according to age group. Nevertheless, by 2006–2007, the campaign had achieved significantly fewer antibiotic prescriptions: rates were −27.0% (95% CI −33.5% to −20.5%) versus baseline for the whole population, −30.1% (95% CI −40.7% to −19.6%) for the youngest children, −35.8% (95% CI −48.3% to −23.2%) for 6- to 15-y-olds, and −20.5% (95% CI −25.6% to −15.4%) for those >15 y (Table 2).

**Table 2:** Estimated mean percent reduction of antibiotic use [95% CI] and associated *p*-values, compared to 2000–2002 baseline values.

Period	Age Group	2002–2003	2003–2004	2004–2005	2005–2006	2006–2007
October–March (winter)	0–5 y (FLS unadjusted)	−1.2 [−7.7 to 5.3]	−2.4 [−10.8 to 5.9]	−14.9 [−24.2 to −5.6]	−26.9 [−36.9 to −17.0]	−30.1 [−40.7 to −19.6]
	*p*-value	0.71	0.87	0.002	<0.0001	<0.0001
	6–15 y (FLS unadjusted)	−5.2 [−14.1 to 3.6]	−15.3 [−26.1 to −4.4]	−26.1 [−37.7 to −14.5]	−26.2 [−38.1 to −14.3]	−35.8 [−48.3 to −23.2]
	*p*-value	0.24	0.006	<0.0001	<0.0001	<0.0001
	>15 y (FLS unadjusted)	−12.5 [−16.8 to −8.1]	−11.3 [−16.2 to −6.5]	−13.4 [−18.4 to −8.5]	−19.0 [−23.9 to −14.0]	−20.5 [−25.6 to −15.4]
	*p*-value	<0.0001	<0.0001	<0.0001	<0.0001	<0.0001
	All (FLS unadjusted)	−9.8 [−14.9 to −4.7]	−10.9 [−16.8 to −4.9]	−16.9 [−23.1 to −10.6]	−23.8 [−30.1 to −17.4]	−27.0 [−33.5 to −20.5]
	*p*-value	0.0002	0.0003	<0.0001	<0.0001	<0.0001
	All (FLS adjusted)^a^	−8.5 [−12.8 to −4.3]	−10.0 [−15.7 to −4.3]	−16.1 [−22.5 to −9.7]	−21.9 [−28.6 to −15.2]	−26.5 [−33.5 to −19.6]
	*p*-value	0.003	0.0002	<0.0001	<0.0001	<0.0001
October–September b	All (FLS unadjusted)	−7.3 [−12.4 to −2.2]	−8.0 [−14.0 to −2.0]	−11.9 [−18.2 to −5.6]	−17.3 [−23.8 to −10.9]	—
	*p*-value	0.005	0.009	0.0002	<0.0001	—

a Adjusted to FLS frequency with interaction term indicating different FLS effects before and after the second campaign.

b After October 2006 data were only available until March 2007, therefore the 2006–2007 period was not taken into account.

As expected, the FLS incidence rate was significantly associated with the weekly rate of antibiotic prescription (*p*<0.0001). However, a significant decrease of 45% in the linear regression coefficient between FLS incidence and antibiotic prescriptions was observed after the first campaign (1.19 before versus 0.66 after, *p* = 0.006). After controlling for the influence of the FLS incidence rate, we observed a −26.5% (95% CI −33.5% to −19.6%) change in winter antibiotic consumption for the total population, a decrease similar to that obtained without controlling for the FLS incidence rate. Including four dummy variables in the model for the April–September period did not show any clear change during summer. From 2003 to 2005, rate changes ranged between −3% to −5%, but were not significant. In 2006, an estimated decrease of −8.5% was marginally significant (*p* = 0.08).

## Discussion

Between 2002 and 2007, we observed an overall 26.5% reduction (adjusted for FLS fluctuations) of winter antibiotic prescriptions in France; the reduction was consistent across all age groups and all 22 regions, as well as across the most frequently used therapeutic classes. The initial objective of the “Keep Antibiotics Working” program to reduce antibiotic use in the community by 25% was reached in 5 years.

The impact of educational campaigns aimed at decreasing antibiotic prescriptions by preventing antibiotic misuse at the population level has rarely been evaluated. In Europe the European Surveillance of Antimicrobial Consumption (ESAC, http://www.esac.ua.ac.be) program collects data on antimicrobial consumption in ambulatory care and hospital settings from 34 European countries' national surveillance systems and publishes a yearly report [23]. In the United States, investigations on antibiotic consumption trends are based on data recorded for a population sample as provided by the National Ambulatory Medical Care Survey [24]–[27]. Other published North American studies evaluated interventional campaigns but are based on data from only a few medical practices [28],[29], Medicaid [30], or other medical care providers [31]–[36]. Few other interventional studies based on national data are available: Belgium [37], Sweden [38], and Australia [39]. Prescription data in North America have also been analyzed in several areas (British Columbia, Wisconsin, and Minnesota) [40],[41].

In the European Union, several countries have initiated national campaigns to optimize antibiotic use [42]. Our results are consistent with the recently published article from the ESAC program; this article similarly reported a reduction of >15% of antimicrobial drug use for penicillins, cephalosporins, and macrolides (similarly, no reduction was noted for quinolones) during the 2000–2004 period [4]. The overall decline we observed can be compared to those observed in three other national studies for which comparable data are available: Belgium, Sweden, and Australia. In Belgium, a significant decrease (after controlling for FLS variation) was obtained after the first year but not after the second [37]; a longer follow-up might show a more pronounced and long-term decrease [43]. In Sweden, a country that had one of the lowest antibiotic consumption rates in Europe [23], an intervention was launched in 1995 [44]. This intervention did not involve a public campaign and was reported as having led to a −20% change in overall antibiotic sales between 1995 and 2004 [38]. The Australian investigation focused mainly on consumer awareness and physician behavior changes [39].

In France, comprehensive coverage of the population by the NHI and drug reimbursement for outpatient care offer a unique opportunity for in-depth analysis of drug use data, in particular use of antibiotics among outpatients for almost the entire population. To the best of our knowledge, this is the largest body of data—with nearly half a billion data entries and the longest time-series of individual and weekly data on antibiotic prescriptions—to evaluate antibiotic use in the community ever analyzed.

Our data show that the primary objective of the French national campaign was largely achieved, with a 30.1% decrease in antibiotic use in children <6 y old. This result is very encouraging, because a substantial proportion of antibiotic prescriptions for young children are unnecessary because of the viral origins of their infection [26]. The most important decrease in antibiotic use for children <5 y was noted after the second campaign. There is no clear explanation for this change, but it suggests that repeated campaigns may be necessary to overcome initial resistance by parents and physicians to reduced antibiotic prescription.

In addition, children aged 6–15 y also had significantly lower antibiotic use (a −35.8% change over the study period). This change may be due to the use of rapid tests to diagnose group A streptococci tonsillitis, which was promoted by the campaign in this age group, which is at a higher risk of group A streptococci throat infections.

Antibiotic consumption changed by −24.1% among young adults (26–35 y), who were initially the biggest antibiotic consumers among adults. Demographic characteristics of this population suggest that most parents of young children fall within this age category; as a result, it is likely that young adults have been specifically affected by the campaign. Finally, prescriptions in the 21–25 y age group remained stable from 2000 to 2007 (as compared to decreases observed in older and younger age groups). Two hypotheses may explain this observation. First, 21- to 25-y-old adults are less likely to live in or interact with collective institutions (daycares, schools, etc.) or be in close contact with children than other age groups; as a result, they are less likely to be exposed to VRIs, which are typically spread by young children. Second, it may be difficult to further decrease antibiotic use in this age group, as they already represent the age group with the lowest antibiotic prescription rate before the campaign.

While crude results did not differ markedly from those adjusted for FLS fluctuations, accounting for the latter was crucial to the interpretation of the changes recorded. Indeed, in many countries, VRIs account for a high share of unnecessary antibiotic prescriptions. Our intervention model highlighted a significant weakening of the association between FLS incidence rates and antibiotic prescriptions after the second yearly campaign. This observation suggests that the fraction of antibiotics prescribed for viral illnesses has significantly decreased, which is a highly encouraging result, as one of the campaign's main objectives was to reduce VRI-associated antibiotic overuse. However, the association between FLS and antibiotic prescriptions persists, at a low level, even after the campaign.

Several limitations of this study should be noted. First, due to the quasiexperimental design (i.e., absence of a control group) and limited preintervention data, a cause–effect relationship between the campaign “Antibiotics are not automatic” and decreased antibiotic use cannot be proved. For example, the influence of the antibiotic campaigns in other geographically proximate European countries such as Belgium cannot be excluded. Some southern European countries also conducted campaigns during the 2002–2007 period (Greece: mass media campaign in 2001–2003; Spain: mass media campaign since 2006; Portugal: radio campaign in 2004–2007) and observed evolution of outpatient antibiotic use [23]. Thus, effects of targeted campaigns versus the spontaneous decrease of antibiotic use should be evaluated in Europe. Very few new antibiotics have been launched in the past decade; as a result, the promotion of antibiotic prescription by the pharmaceutical industry has probably decreased. This might be a confounding factor for the observed reduction of antibiotic use.

Second, we did not have access to information about the pathology for which antibiotics had been prescribed. In France, no information system exists that provides easy access to data linking drug use to a clinical condition.

Third, we did not account for the introduction of the 7-PCV in our analysis. The 7-PCV initially received marketing authorization in France in 2002, specifically for children presenting specific risk factors. It became widely used only at the end of 2006, once it had been recommended for all children <2 y old (unpublished data). It is thus unlikely that its market introduction could explain much of the decreased antibiotic use observed over the 5-year investigation period.

Fourth, FLS surveillance data do not account for other VRIs such as infections due to respiratory syncytial virus, which generally occur several weeks before a flu outbreak. It was not feasible to account for these viral infections, as surveillance data of these infections are not available in France. As a result, it is likely that we underestimated the association between community viral infection and antibiotic prescription.

Fifth, other local initiatives were promoted in France since 2000, such as the campaign “Antibiotics Only When Necessary” promoted in a county in southeastern France (see http://www.gepie.org/). The added value of such initiatives was not specifically investigated.

Sixth, some authors have reported that antibiotics may substantially reduce the risk of pneumonia after chest infection [45]. Therefore, adverse effects of reduced antibiotic use (e.g., increase in certain severe infections) is questionable. We do not address this question here, which remains to be investigated.

Reasons for reduced antibiotic prescriptions, e.g., fewer consultations or improved prescribing, were not evaluated here, the data did not provide the necessary information to address this question. It has been reported that mass media campaigns play a role in influencing antimicrobial prescription practice in the UK [46]. Due to the multifaceted approach and targeting of the general public and physicians in parallel, the individual effect of each approach could not be evaluated. We believe that the success of the intervention was in fact a result of the combined approach, e.g., face-to-face peer education and widespread public campaigns, allowing both the practitioner not to prescribe and the patient not to ask for antibiotic therapy.

The results of the present report are highly promising in terms of bacterial resistance control. A recent European study confirms the ecological relationship between antibiotic consumption and rate of MRP at the national level [4]. This study strongly underlines the responsibility of countries with higher levels of antibiotic use and recommends that they urgently undertake campaigns devoted to the control of MRP, including the promotion of prudent antibiotic use.

In France from 2001 to 2006, a decreasing trend was observed in the rate of pneumococci resistant to penicillin (47% to 32% of isolates) and the rate of pneumococci resistant to macrolides (49% to 36%) in France (see http://www.rivm.nl/earss). Because our campaign did not target any specific therapeutic class, it may have prevented interclass switching. Nevertheless, a slight increase of quinolone prescriptions occurred. Although this was a moderate increase compared to large decreases recorded for all other classes, this trends points to the need for careful monitoring of quinolone-resistant bacteria in the community [47].

Despite the sharp reduction of antibiotic prescriptions observed, France remains a high user of antibiotics [23]. Nevertheless, the impact of the decrease in antibiotic use on the prevalence of infections caused by antimicrobial-susceptible and antimicrobial-resistant strains must be investigated. Future studies should combine the assessment of 7-PCV vaccination and antibiotic-reduction policies, and evaluate their respective role in the evolution of *S. pneumoniae* invasive infections, according to strain susceptibility.

## Supporting Information

Text S1 **Description of the public health campaign “Antibiotics are not automatic” between 2002 and 2007.**
(0.04 MB DOC)

## Abbreviations

7-PCV: 7-valent pneumococcal conjugate vaccine
ARMA: autoregressive and moving average
ATC: anatomical therapeutic class
CI: confidence interval
FLS: flu-like syndrome
MRP: multidrug-resistant pneumococci
NHI: [French] National Health Insurance
VRI: viral respiratory infection
