# Supplementary material for: Significant Reduction of Antibiotic Use in the Community after a Nationwide Campaign in France, 2002–2007
Source: PLoS Med. 2009 Jun 2;6(6):e1000084. doi: 10.1371/journal.pmed.1000084 (PMC2683932; doi:10.1371/journal.pmed.1000084)
Supplement: Text S1 — Description of the public health campaign “Antibiotics are not automatic” between 2002 and 2007. (0.04 MB DOC) [file pmed.1000084.s001.doc]

## Text S1. Description of the public health campaign “Antibiotics are not automatic” between 2002-2007

The campaign mainly focused on the unnecessary use of antibiotics in VRI such as the common cold, rhinopharyngitis, bronchitis and pharyngitis. It targeted two main audiences: healthcare professionals and the general population. The message "Antibiotics are not automatic" was aimed at modifying behavior automatism for treatment of VRI with antibiotics. This is consistent with the conclusion of the 2002 International Forum on Antibiotic Resistance colloquium [1].

An educational campaign was initiated for healthcare workers (HCW), (e.g. 54,000 general practitioners (GPs), 2,700 pediatricians in community practice, and daycare workers, using face-to-face education and guideline dissemination. In addition to providing information on appropriate antibiotic use, the campaign promoted the use of rapid tests to diagnose group A streptococci (RTAS) tonsillitis. Physicians were invited to county-level trainings on how to use RTAS and optimize antibiotic use. They were also visited by NHI physicians for face-to-face peer education sessions. Free tests were provided to physicians who attended training. Antibiotic guidelines were sent to targeted practitioners and distributed during trainings and visits. Among nearly 2,400,000 children <3 years, one quarter attend daycare centers. Daycare workers were trained to not systematically require antibiotic treatment as a condition for daycare attendance of children with VRI as well as to relay the message of optimal use of antibiotics to parents.

The public campaign mainly targeted children and their parents, underlining that (1) antibiotic efficacy is endangered by bacterial resistance and it is essential to preserve their efficacy; (2) higher consumption rates are linked to higher resistance levels; (3) antibiotics do not cure VRI; (4) the majority of upper respiratory infection are of viral origin; (5) antibiotics do not lower fever, lessen pain, or shorten duration of illness; and (6) it is important to fully respect treatment duration and dosage prescribed. The general public campaign appeared in national media outlets, consisting of prime-time television and radio ads, newspaper ads and a web site, and in physician’s offices, including booklets, handouts and posters in physicians’ waiting rooms. A traveling educational exhibit about microbes was also created. Some of the educational materials used in the campaign are available at [http://www.antibiotiquespasautomatiques.com](http://www.antibiotiquespasautomatiques.com/).

An analysis of NHI campaign data has estimated that 8 million RTAS were distributed to physicians and pediatricians between 2002 and 2007. Furthermore, 45,000 GPs had at least one peer visit with an NHI physician between 2003 and 2005. From 2002 to 2008, the cost of the campaign was estimated to be 500,000,000€, *i.e*. ~1.40€ per inhabitant per year. Between 2002 and 2007, direct savings related to decreased antibiotic use was estimated to be 850,000,000€, *i.e* ~2.80€ per inhabitant per year.

NHI also undertook a follow-up survey of physician and general public knowledge and attitudes. It was reported that parents expected an antibiotic prescription during 37% of VRI consultations in 2002 and during 23% of VRI consultations in 2007. Thirty percent of people knew that antibiotics are only effective against bacteria in 2002, but this figure rose to 40% in 2007. Eight out of ten physicians were more confident in their decisions not to prescribe unnecessary antibiotics in 2007 [2].

Since 2007, the French campaign has undertaken a new communication phase; it attempts to better inform consumers and to link VRI with the absence of antibiotic prescription. This program will continue for at least the four years: 2008-2012.

References

1. Finch RG, Metlay JP, Davey PG, Baker LJ (2004) Educational interventions to improve antibiotic

use in the community: report from the International Forum on Antibiotic Resistance (IFAR)

colloquium, 2002. Lancet Infect Dis 4: 44-53.

2. Chapuis G. The French Public Campaigns "Antibiotics, it's not automatic". European

Worksop"Public awareness campaigns on the prudent use of antibiotics. French Health Ministry;

2008; Paris. (www.sante-jeunesse-sports.gouv.fr/dossiers/sante/antibiotiques/atelier-europeen-6-7-

novembre-2008.html)
